# The Validity and Reliability of the Mini-Mental State Examination-2 for Detecting Mild Cognitive Impairment and Alzheimer’s Disease in a Korean Population

**DOI:** 10.1371/journal.pone.0163792

**Published:** 2016-09-26

**Authors:** Min Jae Baek, Karyeong Kim, Young Ho Park, SangYun Kim

**Affiliations:** 1 Clinical Neuroscience Institute, Seoul National University Bundang Hospital, Seoungnam-si, Gyeonggi-do, Republic of Korea; 2 Department of Translational Medicine, Seoul National University College of Medicine, Seoul, Republic of Korea; 3 Department of Neurology, Seoul National University College of Medicine, Seoul, Republic of Korea; Banner Alzheimer's Institute, UNITED STATES

## Abstract

**Objective:**

To examine the validity and reliability of the MMSE-2 for assessing patients with mild cognitive impairment (MCI) and Alzheimer’s disease (AD) in a Korean population. Specifically, the usefulness of the MMSE-2 as a screening measure for detecting early cognitive change, which has not been detectable through the MMSE, was examined.

**Methods:**

Two-hundred and twenty-six patients with MCI, 97 patients with AD, and 91 healthy older adults were recruited. All participants consented to examination with the MMSE-2, the MMSE, and other detailed neuropsychological assessments.

**Results:**

The MMSE-2 performed well in discriminating participants across Clinical Dementia Rating (CDR) stages and CDR-Sum of Boxes (CDR-SOB), and it showed excellent internal consistency, high test-retest reliability, high interrater reliability, and good concurrent validity with the MMSE and other detailed neuropsychological assessments. The MMSE-2 was divided into two factors (tests that are sensitive to decline in cognitive functions vs. tests that are not sensitive to decline in cognitive functions) in normal cognitive aging. Moreover, the MMSE-2 was divided into two factors (tests related overall cognitive functioning other than memory vs. tests related to episodic memory) in patients with AD. Finally, the MMSE-2 was divided into three factors (tests related to working memory and frontal lobe functioning vs. tests related to verbal memory vs. tests related to orientation and immediate recall) in patients with MCI. The sensitivity and specificity of the three versions of the MMSE-2 were relatively high in discriminating participants with normal cognitive aging from patients with MCI and AD.

**Conclusion:**

The MMSE-2 is a valid and reliable cognitive screening instrument for assessing cognitive impairment in a Korean population, but its ability to distinguish patients with MCI from those with normal cognitive aging may not be as highly sensitive as expected.

## Introduction

The dementia prevalence rate among elderly people is rapidly increasing as the general population of most countries age. Early detection is the best way to treat dementia and to plan healthcare. Although the Diagnostic and Statistical Manual of Mental Disorder (DSM-5) criteria [1] are used for the diagnosis of dementia, screening tests can identify patients at risk.

The Mini-Mental State Examination (MMSE) is the one of the most widely used screening tests in clinical trials and in general practice to detect cognitive impairment in older adults [2, 3]. The MMSE is a quick and easy measure that assesses seven areas of cognitive functioning, and it was shown to have both good test-retest reliability (0.80–0.95) [2–5] and acceptable sensitivity and specificity to detect mild to moderate stages of dementia [2–7]. However, the MMSE is less sensitive in detecting patients with mild cognitive impairment (MCI) and those in the early stages of dementia, and it is also insensitive to impairments in executive functioning, abstract reasoning, and visual perception/construction [8–10]. Moreover, false-positive errors might be more common among patients with less education and of lower socioeconomic status, and a ceiling effect might be more common among patients with a high level of education and patients with MCI because of the low level of item difficulty [11]. Furthermore, some items in the MMSE were difficult to translate into another language, so they have been adjusted to accommodate the culture of each country.

These limitations led to the development of the Mini-Mental State Examination, 2^nd^ edition (MMSE-2) [12] as a reliable cognitive screening measure to provide finer discrimination. First, there are equivalent, alternative forms of each MMSE-2 version (red and blue forms) to decrease the possibility of practice effects that can occur over serial examinations. The equivalency of the alternative MMSE-2 forms was 0.96 [12]. Moreover, unlike the MMSE, there are three different versions of the MMSE-2: the MMSE-2: Brief Version (MMSE-2:BV), which is a shortened version of the MMSE; the MMSE-2: Standard Version (MMSE-2:SV), which is equivalent to the MMSE; and the MMSE-2: Expanded version (MMSE-2:EV), which is slightly longer than the MMSE, is more sensitive to changes with aging and has a ceiling effect.

The total score of the MMSE-2: BV is 16 points. This test is simpler than the MMSE, and it is used to conduct a rapid clinical assessment and to screen larger populations. The MMSE-2:BV is comprised of four items: registration, orientation to time, orientation to place, and recall. According to Folstein et al. [12], these four items have adequate sensitivity and specificity to detect the cognitive decline of patients with dementia.

The total possible score on the MMSE-2:SV, is 30 points, which is the same as the total score on the MMSE. The structure of the MMSE was maintained, but some items from the MMSE were changed. Among them, some items that were hard to translate into other languages were changed, and some items were replaced to increase the degree of difficulty of the MMSE-2.

Finally, the total score of the MMSE-2:EV is 90 points because two more items (story memory and processing speed) were added to increase the clinical utility of the MMSE by extending a ceiling effect and to increase the sensitivity and specificity of this version to detect cognitive impairment not only in patients with Alzheimer’s disease (AD) but also in patients with subcortical dementia. The story memory item evaluates verbal explicit learning and verbal free recall, and the processing speed test (symbol-digit-coding test) measures psychomotor ability and incidental learning primarily associated with the executive function of the frontal lobe [12].

According to the result of the study on the MMSE-2 [12], the sensitivities of the MMSE-2:SV and the MMSE-2:EV were both 84% for discriminating patients with AD from healthy older adults. Moreover, the sensitivities of the MMSE-2:SV and the MMSE-2:EV were 72% and 75% for discriminating patients with subcortical dementia from normal cognitive aging. That is, the MMSE-2 is a more useful screening measure for dementia and cognitive impairment than the MMSE.

Therefore, the purpose of this study is to evaluate the validity and reliability of the MMSE-2 for assessing patients with MCI and AD in a Korean population. Specifically, we would like to focus on the usefulness of the MMSE-2 as a sensitive screening measure for detecting early cognitive change, which has not been detectable via the MMSE.

## Materials and Methods

### Ethical Issue

This study was approved by the Seoul National University Hospital Institutional Review Board of each participating site and written informed consent was obtained from all subjects before all procedures. In case of the AD patients who had impaired ability to consent, written consents were obtained from the care giver on their behalf. The IRV approval number is “B-1306/208-105”.

### Participants

#### Patients

Between June 2012 and April 2013, 323 outpatients and inpatients at the Clinical Neuroscience Center at the Seoul National University Bundang Hospital who complained of memory disturbance or a decline in cognitive functioning underwent a medical examination via an interview, a neurological examination, blood tests, brain imaging with CT or MRI, and neuropsychological assessments to obtain a diagnosis. Among them, 226 patients (90 male, 136 female) were diagnosed with MCI, and 97 patients (36 male, 61 female) were diagnosed with AD. All patients were over 50 years old.

The patients with MCI were diagnosed according to Petersen’s criteria [13], and the patients with AD were diagnosed with ‘probable AD’ based on the criteria of the National Institute of Neurological and Communicative Disorders and Stroke and the Alzheimer’s disease and Related Disorders Associations (NINCDS-ADRDA) [14]. Moreover, based on the Clinical Dementia Rating (CDR) [15] and the Clinical Dementia Rating Scale Sum of Boxes (CDR-SOB) [16, 17] scores, patients were classified as having MCI (CDR 0.5, CDR-SOB 0.5–2.5), early stage of AD (CDR 0.5, CDR-SOB 3.0–4.0), mild stage of AD (CDR 1, CDR-SOB 4.5–9.0), or moderate stage of AD (CDR 2, CDR-SOB 9.5–15.5).

#### Control participants

Between June 2012 and April 2013, 91 healthy adults who were all over 50 years of old age participated in this study. They were either the caregivers for one of the patients undergoing treatment at Seoul National University Bundang Hospital, or they were recruited from a health care center. They did not have subjective memory complaints, any of 29 exclusionary diseases, or a history suggestive of a decrease in cognitive function [18]. They also had scores that were higher than or at most one standard deviation below the mean scores of the respective age- and education-matched population on the Mini-Mental State Examination in Korea [9] and had an average score of 0.42 or lower on the Korean Instrumental Activities of Daily Living (K-IADL) [19]. This score has been found to discriminate dementia from normal cognitive aging. The K-IADL is an 11-item questionnaire that includes IADLs of shopping, mode of transportation, ability to handle finances, housekeeping, food preparation, ability to use a telephone, taking medication, recent memory, hobbies, watching television, and fixing. All participants were determined to be free of cognitive deficits, and they all consented to participate in this study. Moreover, all participants were free from neurological or psychiatric illnesses, underwent the same neuropsychological assessments as the cognitively impaired subjects and were included in the healthy control group.

### Instruments

#### MMSE-2

The Psychological Association Research (PAR) holds the copyright to the MMSE-2, and they allowed us to translate the MMSE-2 into Korean before the start of this study. Because one of the purposes of developing the MMSE-2 was to conduct the same test across the world, which the MMSE cannot do, the items of the MMSE-2 should not be modified to adjust to cultural background. Therefore, two neurologists and two neuropsychologists with over ten years of clinical experience translated the MMSE-2 into Korean and then, after performing many stages of modification, sent the back-translated MMSE-2 to PAR for certification. The final version of the MMSE-2 in Korean was modified by experts at PAR, and then the Korean version of the MMSE-2 was finalized in April 2012.

The MMSE-2 is composed of alternative forms, such as the red form and the blue form, to reduce the learning effect that may take place upon repeated use. Moreover, as mentioned earlier, the MMSE-2 has three versions, the MMSE-2:BV, the MMSE-2:SV, and the MMSE-2:EV, and nine subtests of the MMSE-2 are as follows.

The MMSE-2:BV is composed of four subtests in the following order: registration, orientation to time, orientation to place, and recall. The MMSE-2:SV is composed of seven subtests in the following order: attention and calculation, language, drawing, and the four subtests of the MMSE-2:BV. The MMSE-2:EV is composed of nine subtests in the following order: story memory, processing speed, and the seven subtests of the MMSE-2:SV. The detailed explanation of story memory and processing speed, which are included in the MMSE-2, is as follows.

#### Story memory test

The story memory test measures verbal explicit learning and verbal immediate free recall. The story memory test is composed of four sentences, and the sentences are on a sixth grade reading level. The red form and the blue form of the MMSE-2 each contain a different story. The story on the red form was 62 words long, and the story on the blue form was 66 words long. Each story was written in the past tense using the active voice, and contained no repetitive words or phrases [12].

#### Processing speed test (Symbol-Digit-Coding test)

The processing speed measure is tapping into frontal lobe areas. This test measures psychomotor ability primarily associated with executive function of the frontal lobe: this is one of the components of the symbol-digit-coding test. Participants are asked to pair symbols with digits within a 30 seconds time limit. Although the stimuli of the red and blue forms are the same, the template that the participants draw is different on each form of the test [12].

#### Other neuropsychological assessments

To measure the correlation of the MMSE-2 with other neuropsychological assessments, a variety of cognitive functions, such as attention, verbal memory, visuospatial function ability, executive function, and language function, were measured. Attention was assessed using forward and backward digit span tests [20]. Verbal memory was assessed using the Seoul Verbal Learning Test (SVLT) [21], and a copy of the Rey Complex Figure Test (RCFT) [22] was used to assess visuospatial function. Neuropsychological assessments primarily associated with executive function, including the Stroop Color-Word Test [23], the Semantic Word Fluency Test (SWF) and the Phonemic Word Fluency Test (PWF) [24], were used. Naming ability was assessed using the Korean version of the Boston Naming Test (K-BNT) [25]. Global measurements, including the MMSE in Korean [9], CDR [15], and CDR-SOB [16, 17], were also conducted.

### Procedure

First, to measure the equivalency of the alternative forms of the MMSE-2 tests, 138 patients completed both the red and blue forms of the MMSE-2. To eliminate the order effect, half of the 138 patients completed the red form of the MMSE-2 first and the other half completed the blue form of the MMSE-2 first. According to the result of Pearson’s correlation analysis, the correlation coefficient was high between the red and blue forms of the MMSE-2:BV (r = 0.90, *p*<0.001), the MMSE-2:SV (r = 0.97, *p*<0.001), and the MMSE-2:EV (r = 0.97, *p*<0.001).

Therefore, half of the patients who participated in this study completed the red form of the MMSE-2, and the other half of completed the blue form of the MMSE-2. Also, the order in which the neuropsychological assessments were administered is shown in Table 1.

**Table 1:** Order of neuropsychological assessments.

Order	List of neuropsychological assessments
1	MMSE-2 (red form or blue form)
2	SVLT-immediate recall
3	RCFT-copy
4	Digit span-forward
5	Digit span-backward
6	Stroop Color-Word test (word reading)
7	Stroop Color-Word test (color naming)
8	SVLT-delayed recall
9	SVLT-recognition
10	SWF-animal
11	SWF-supermarket items
12	PWF-three Korean alphabets
13	K-BNT
14	MMSE

Abbreviations: MMSE-2, Mini-Mental State Examination-2; SVLT, Seoul Verbal Learning Test; RCFT, Rey Complex Figure Test; SWF, Semantic Word Fluency; PWF, Phonemic Word Fluency; K-BNT, Korean version of Boston Naming Test; MMSE, Korean version of the Mini-Mental State Examination.

### Statistical analysis

An analysis of variance (ANOVA) was used to compare age and education levels, and a chi-square test was used to compare gender across all three groups. The results of the neuropsychological tests including the MMSE, among the three groups were analyzed using an analysis of covariance (ANCOVA) after controlling for demographic variables (age and education). Moreover, the MMSE-2 scores of all three groups were analyzed with an ANCOVA followed by Tukey’s test for *post-hoc* analysis.

Reliability was assessed through measurements of internal consistency, test-retest reliability, and interrater reliability. The internal consistency of the MMSE-2 was measured using Cronbach’s α coefficient. To assess test-retest reliability, the MMSE-2 was re-administered one to two months (34.48±3.48 days) after the initial test to 16 patients with MCI, 4 patients with AD, and 7 healthy older adults, and the data were analyzed using Pearson’s correlation coefficients. Moreover, to assess the equivalency of the blue and red forms of the MMSE-2, 138 participants were given both the blue and red forms in a counterbalanced design, with the second administration immediately following the first, and the data were analyzed using Pearson correlation coefficients. Interrater reliability was calculated between two neuropsychologists (n = 160) using the intraclass correlation coefficient (ICC).

Finally, the validity of the MMSE-2 was analyzed as follows. To evaluate the construct validity, a Varimax rotated factor analysis was used to explore the factor structure of the 13 items. Moreover, to assess the concurrent validity of the MMSE-2, Pearson’s correlation coefficient was used to compare the MMSE-2 with the MMSE, the CDR, the CDR-SOB, SVLT, the copy test of RCFT, the SWF, the PWF, the Stroop Color-Word test, the K-BNT, and the digit span test (forward & backward). To verify the discriminant validity based on the severity of dementia, all participants were classified into four groups according to CDR and CDR-SOB, and the average scores of the MMSE-2 were compared among these four groups using ANCOVA. To evaluate the diagnostic utility of the MMSE-2, the sensitivity and specificity of the MMSE-2 was examined using a receiver operating characteristics (ROC) curve and area under the curve (AUC) measurements. Data were analyzed using SPSS 18.0. (SPSS Inc., Chicago, IL, USA), and *p*<0.05 was considered to be significant for all analyses.

## Results

### The participants’ demographic data

The demographic data are presented in Table 2. A total of 414 elderly participants (155 men and 259 women) were enrolled in this study. The mean age of the patients with MCI was 71.05±7.73 years (range: 70–72 years), and the mean age of the patients with AD was 75.38±7.60 (range: 73–77 years). The mean age of the healthy older adults was 67.05±7.55 years (range: 65–69 years). The mean number of years of education was 11.45±4.80 years (range: 10–12 years) in the patients with MCI, 9.63±5.15 years (range: 8–11 years) in the patients with AD, and 10.98±5.21 years (range: 9–12 years) in the healthy older adults.

**Table 2:** Characteristics of participants (M±SD).

	All participants (n = 414)		
	Normal (n = 91)	MCI (n = 226)	AD (n = 97)
Age (years)	67.05±7.55	71.05±7.73*	75.38±7.60†
Education (years)	10.98±5.21	11.45±4.80	9.63±5.15‡
Male/Female	29/62	90/136	36/61

Abbreviations: M, Mean; SD, Standard deviation; MCI, Mild Cognitive impairment; AD, Alzheimer’s disease.

Note.

*p<0.001 for MCI vs. Normal.

†p<0.001 for AD vs. MCI and normal.

‡p<0.013 for AD vs. MCI.

There was no significant difference in the participants’ gender, **χ**^2^ (1,414) = 1.76, *p* = 0.42, but there were significant differences in age, F(2, 411) = 27.82, *p*<0.001, and education, F(2, 411) = 4.38, *p*<0.013, between the three groups. According to Tukey’s *post hoc* analysis, the mean age of the patients with AD was significantly higher than the mean age of the patients with MCI and of the healthy older adults, and the mean age for the patients with MCI was significantly higher than that of the healthy older adults. Moreover, the mean number of years of education for the patients with MCI was higher than for the patients with AD, and there was no significant difference in the mean number of years of education between the patients with AD and the healthy older adults or between the patients with MCI and the healthy older adults.

### The results of participants’ neuropsychological assessments

The results of the neuropsychological assessments of the three groups (MCI, AD, and healthy older adults) were compared. With respect to each of the cognitive domain scores, the three groups differed significantly in each of the domain assessed: attention, verbal memory, visuospatial function, language function, and frontal/executive function (all *p*<0.05). Tukey’s *post hoc* analysis of the cognitive domain revealed that the scores of the healthy older adults were significantly higher than the scores of the patients with MCI and AD and that the scores of the patients with MCI were significantly higher than the scores of the patients with AD in the MMSE, the SVLT, the copy of the RCFT, the SWF, the PWF, the Stroop Color-Word test (color naming), the K-BNT, and the digit span test (forward & backward). However, in the Stroop Color-Word test (word reading), although there was no significant difference between the healthy older adults and the patients with MCI, the scores of the two groups (healthy older adults and MCI) were significantly higher than those of the patients with AD. The mean scores of the subtests for each group and the results of Tukey’s *post-hoc* analysis are presented in Table 3.

**Table 3:** The results of neuropsychological assessments in the three groups (M±SD).

Neuropsychological assessments	C	MCI	AD	F	*df*	*Post-hoc*
MMSE	27.29±2.31	25.68±2.68	19.33±3.82	201.32*	2, 409	C>M>A
SVLT-immediate recall	21.47±4.06	17.15±4.39	10.91±3.71	111.87*	2, 409	C>M>A
SVLT-delayed recall	7.26±2.00	3.49±2.92	0.29±0.92	152.14*	2, 409	C>M>A
SVLT-recognition	9.46±1.68	7.42±2.42	3.70±2.87	104.73*	2,409	C>M>A
RCFT-copy	32.41±3.87	28.87±5.32	22.50±7.63	66.91*	2, 403	C>M>A
SWF-animal	17.40±4.14	12.86±3.75	8.84±4.31	80.74*	2, 409	C>M>A
SWF-supermarket items	18.97±5.32	14.46±5.84	8.33±4.57	61.57*	2, 404	C>M>A
PWF-ㄱ, ㅇ, ㅅ	26.35±11.01	20.41±9.78	13.83±8.95	27.51*	2, 358	C>M>A
Stroop Color-Word test (word reading)	110.89±5.98	111.13±4.03	105.31±13.68	13.49*	2, 374	C = M>A
Stroop Color-Word test (color naming)	88.30±17.91	70.41±23.45	40.73±24.30	63.86*	2, 354	C>M>A
K-BNT	48.15±8.09	40.48±9.97	28.70±11.22	63.33*	2, 409	C>M>A
Digit span-forward	6.07±1.51	5.63±1.40	4.91±1.45	6.44*	2, 409	C>M>A
Digit span-backward	4.10±1.15	3.56±0.97	2.92±0.95	25.23*	2, 409	C>M>A

Abbreviations: M, Mean; SD, Standard deviation; C, Control; MCI, Mild Cognitive Impairment; AD, Alzheimer's Disease; MMSE, Korean version of the Mini-Mental State Examination; SVLT, Seoul Verbal Learning Test; RCFT, Rey Complex Figure Test; SWF, Semantic Word Fluency; PWF, Phonemic Word Fluency; K-BNT, Korean version of Boston Naming Test.

Note.

*p< 0.05.

### MMSE-2

#### The equivalency of the blue and red forms of the MMSE-2

The MMSE-2 blue and red forms equating sample consisted of 138 participants with an average age of 72.22±7.46 years and an average educational level of 10.58±5.10 years. The average scores of the MMSE-2:BV (red and blue forms), MMSE-2:SV (red and blue forms), and MMSE-2:EV (red and blue forms) are presented in Table 4. The reliability was high for all three alternative forms: the MMSE-2:BV (r = 0.90, *p*<0.01), the MMSE-2:SV (r = 0.97, *p*<0.01), and MMSE-2:EV (r = 0.97, *p*<0.01).

**Table 4:** The results of red and blue forms of the three versions of the MMSE-2 (M±SD).

MMSE-2	Form	M	SD	Alternating forms reliability
Brief Version	Red	12.10	2.896	r = 0.90
	Blue	11.88	2.891	(*p*<0.01)
Standard Version	Red	23.91	4.175	r = 0.97
	Blue	23.82	4.026	(*p*<0.01)
Expanded Version	Red	39.99	11.136	r = 0.97
	Blue	41.12	11.488	(*p*<0.01)

Abbreviations: M, Mean; SD, Standard deviation; MMSE-2, Mini-Mental State Examination-2.

#### The results of the MMSE-2 in the three groups

The MMSE-2:BV scores of the participants in the three groups are shown in Table 5. An ANCOVA that controlled for age and education revealed significant differences between the three groups on the MMSE-2:BV. According to Tukey’s *post hoc* analyses, the total score of the MMSE-2:BV was significantly higher for the healthy older adults than for the patients with MCI and the patients with AD, and it was significantly higher for the patients with MCI than for the patients with AD. Especially, among all items of the MMSE-2, the score of recall was significantly higher for the healthy older adults than for the patients with MCI and the patients with AD, and it was significantly higher for the patients with MCI than for the patients with AD. However, there were no significant differences in the items registration, orientation to time, and orientation to place between the healthy older adults and the patients with MCI, but the scores of the three items in the MMSE-2:BV were significantly higher for the healthy older adults and the patients with MCI than for the patients with AD.

**Table 5:** The results of the MMSE-2:BV in the three groups (M±SD).

MMSE-2:BV	C	MCI	AD	F	*df*	*Post-hoc*
Registration	2.91±0.44	2.91±0.35	2.60±0.59	12.38*	2, 409	C = M>A
Orientation to time	4.80±0.43	4.54±0.77	2.40±1.52	175.53*	2, 409	C = M>A
Orientation to place	4.86±0.38	4.73±0.53	3.62±1.06	91.73*	2, 409	C = M>A
Recall	1.87±0.85	1.20±0.84	0.26±0.51	77.30*	2, 409	C>M>A
**Total score**	14.43±1.32	13.37±1.58	8.91±2.54	230.62*	2, 409	C>M>A

Abbreviations: M, Mean; SD, Standard deviation; C, Control; MCI, Mild Cognitive Impairment; AD, Alzheimer's Disease; MMSE-2:BV, Mini-Mental State Examination-2: Brief Version.

Note.

*p<0.01.

The MMSE-2:SV scores of the participants in the three groups are shown in Table 6. An ANCOVA that controlled for age and education revealed significant differences between the three groups on the MMSE-2:SV. Tukey’s *post hoc* analyses showed that the total score of the MMSE-2:SV was significantly higher for the healthy older adults than for the patients with MCI and the patients with AD, and it was also significantly higher for the patients with MCI than for the patients with AD. Particularly, among all MMSE-2 items, the score of recall was significantly higher for the healthy older adults than for the patients with MCI and the patients with AD, and it was also significantly higher for the patients with MCI than for the patients with AD. However, there were no significant differences in the items registration, orientation to time, orientation to place, attention and calculation, language, or drawing between the healthy older adults and the patients with MCI, but the scores of six items on the MMSE-2:SV were significantly higher for the healthy older adults and the patients with MCI than for the patients with AD.

**Table 6:** The Results of the MMSE-2:SV (M±SD).

MMSE-2:SV	C	MCI	AD	F	*df*	*Post-hoc*
Registration	2.91±0.44	2.91±0.35	2.60±0.59	12.38*	2, 409	C = M>A
Orientation to time	4.80±0.43	4.54±0.77	2.40±1.52	175.53*	2, 409	C = M>A
Orientation to place	4.86±0.38	4.73±0.53	3.62±1.06	91.73*	2, 409	C = M>A
Recall	1.87±0.85	1.20±0.84	0.26±0.51	77.30*	2, 409	C>M>A
Attention and Calculation	4.09±1.17	3.77±1.24	2.61±1.56	28.29*	2, 409	C = M>A
Language	7.69±0.92	7.67±0.57	7.19±1.00	7.94*	2, 409	C = M>A
Drawing	0.95±0.23	0.90±0.30	0.69±0.47	13.54*	2, 409	C = M>A
**Total Score**	27.26±2.66	25.71±2.35	19.39±3.94	205.00*	2, 409	C>M>A

Abbreviations: M, Mean; SD, Standard deviation; C, Control; MCI, Mild Cognitive Impairment; AD, Alzheimer's Disease; MMSE-2:SV, Mini-Mental State Examination-2: Standard Version.

Note.

*p<0.01.

The MMSE-2:EV scores of the participants in the three groups are shown in Table 7. An ANCOVA that controlled for age and education revealed significant differences between the three groups on the MMSE-2:EV. Tukey’s *post hoc* analyses showed that the total score of the MMSE-2:EV was significantly higher for the healthy older adults than for the patients with MCI and the patients with AD, and it was also significantly higher for the patients with MCI than for the patients with AD. Particularly, among the MMSE-2:EV items, the scores of recall, story memory, and processing speed were significantly higher for the healthy older adults than for the patients with MCI and the patients with AD, and the scores of three items were also significantly higher for the patients with MCI than for the patients with AD. However, there were no significant differences in the items registration, orientation to time, orientation to place, attention and calculation, language, or drawing between the healthy older adults and the patients with MCI, but the scores of six items on the MMSE-2:EV were significantly higher for the healthy older adults and the patients with MCI than for the patients with AD.

**Table 7:** The results of the MMSE-2:EV in the three groups (M±SD).

MMSE-2:EV	C	MCI	AD	F	*df*	*Post-hoc*
Registration	2.91±0.44	2.91±0.35	2.60±0.59	12.38*	2, 409	C = M>A
Orientation to time	4.80±0.43	4.54±0.77	2.40±1.52	175.53*	2, 409	C = M>A
Orientation to place	4.86±0.38	4.73±0.53	3.62±1.06	91.73*	2, 409	C = M>A
Recall	1.87±0.85	1.20±0.84	0.26±0.51	77.30*	2, 409	C>M>A
Attention and calculation	4.09±1.17	3.77±1.24	2.61±1.56	28.29*	2, 409	C = M>A
Language	7.69±0.92	7.67±0.57	7.19±1.00	7.94*	2, 409	C = M>A
Drawing	0.95±0.23	0.90±0.30	0.69±0.47	13.54*	2, 409	C = M>A
Story memory	10.46±3.50	7.15±3.21	3.34±1.80	109.96*	2, 409	C>M>A
Processing speed	12.24±4.31	10.47±4.03	6.35±3.40	38.78*	2, 409	C>M>A
**Total Score**	49.84±9.59	43.48±7.81	29.06±7.17	168.37*	2, 409	C>M>A

Abbreviations: M, Mean; SD, Standard deviation; C, Control; MCI, Mild Cognitive Impairment; AD, Alzheimer's Disease; MMSE-2:EV, Mini-Mental State Examination-2: Expanded Version.

Note.

*p<0.01.

### Reliability analyses

#### Internal Consistency

The internal reliability (Cronbach’s α) of three versions of the MMSE-2 (red and blue forms) among the three groups are presented in Table 8. The interrater reliability was high because alphas ranged from 0.62 to 0.79.

**Table 8:** Internal Consistency: MMSE-2:BV, MMSE-2:SV, and MMSE-2:EV (red and blue forms).

MMSE-2	Red form			Blue form		
	C	MCI	AD	C	MCI	AD
BV	0.728	0.730	0.715	0.697	0.718	0.746
SV	0.741	0.665	0.726	0.686	0.676	0.709
EV	0.686	0.698	0.726	0.621	0.668	0.705
Total	0.783	0.747	0.729	0.793	0.751	0.728

Abbreviations: C, Control; MCI, Mild Cognitive Impairment; AD, Alzheimer's Disease; MMSE-2, Mini-Mental State Examination-2; BV, Brief version; SV, Standard version; EV, Expanded version.

#### Test-retest reliability

Sixteen patients with MCI, 4 patients with AD, and 7 healthy older adults were tested twice, at an interval that averaged 34.48±3.48 days, to examine the test-retest reliability. The mean age of the participants was 68.37±11.17 years and the mean number of years of education was 11.17±3.95 years. The test-retest reliability of three versions of the MMSE-2 was high, ranging from 0.76 to 0.90 (Table 9).

**Table 9:** Test-retest reliability of MMSE-2.

MMSE-2		1^st^ Test		2^nd^ Test	
	r	M	SD	M	SD
BV	0.755*	13.48	1.451	13.52	1.553
SV	0.822*	26.04	2.047	26.07	2.147
EV	0.898*	45.67	6.391	44.37	5.871

Abbreviations: M, Mean; SD, Standard deviation; MMSE-2, Mini-Mental State Examination-2; BV, Brief version; SV, Standard version; EV, Expanded version.

Note.

*p<0.01.

#### Interrater reliability

Two trained neuropsychologists were present during the administration of the MMSE-2 to 160 participants. One-way, single-measure intraclass correlation coefficients (ICCs) were calculated for each item of the MMSE-2 (Table 10). The ICCs ranged from 0.94 to 0.99. There was 100% agreement for registration, orientation to time, orientation to place, attention and calculation, naming, repetition, comprehension, reading, writing, drawing, and the psychomotor speed task.

**Table 10:** Interrater reliability of the MMSE-2.

MMSE-2	ICC	% agreement
Registration	-	100%
Orientation to time	-	100%
Orientation to place	-	100%
Recall	0.99	
Attention and calculation	-	100%
Naming	-	100%
Repetition	-	100%
Comprehension	-	100%
Reading	-	100%
Writing	-	100%
Drawing	-	100%
Story memory	0.94	
Processing speed	-	100%

Abbreviations: MMSE-2, Mini-Mental State Examination-2; ICC, Intraclass Correlation Coefficient.

### Validity analyses

#### Construct validity

Construct validity was examined via principal component analysis with Varimax rotation to determine the factor structure of the MMSE-2 in each group. The results of the factor analyses in each group are as follows.

The factor analysis of the healthy older adults identified two factors in the MMSE-2 that explained approximately 48.4% of the total variance, as shown in Table 11. Factor 1 included six subtests (recall, orientation to place, story memory, processing speed, attention and calculation, and orientation to time) that explained 36.0% of the variance. Factor 2 included three subtests (registration, language, and drawing) that explained 12.4% of the variance.

**Table 11:** Factor analysis after Varimax rotation for the control group.

Variables	Factors	
	1	2
Recall	**0.736**	-0.021
Orientation to place	**0.633**	0.076
Story memory	**0.628**	0.432
Processing speed	**0.597**	0.496
Attention and calculation	**0.574**	0.505
Orientation to time	**0.560**	0.029
Registration	0.105	**0.793**
Language	0.045	**0.657**
Drawing	0.060	**0.498**

The factor analysis of the patients with MCI identified three factors in the MMSE-2 that explained approximately 52.2% of the total variance (Table 12). Factor 1 included three subtests (attention and calculation, drawing, and processing speed) that explained 27.5% of the variance. We named this factor “frontal lobe function tests”. Factor 2 included two subtests (recall and story memory) that explained 13.3% of the variance. We named this factor “verbal memory tests”. Factor 3 included three subtests (orientation to place, orientation to time, and registration) that explained 11.3% of the variance. We named this factor “orientation tests”.

**Table 12:** Factor analysis after Varimax rotation for the patients with mild cognitive impairment.

Variables	Factors		
	1	2	3
Attention and Calculation	**0.766**	0.174	-0.228
Drawing	**0.643**	-0.129	0.306
Processing speed	**0.593**	0.271	0.325
Language	0.303	0.291	0.131
Recall	-0.018	**0.839**	0.048
Story memory	0.415	**0.669**	0.124
Orientation to place	0.099	0.149	**0.640**
Orientation to time	-0.168	0.404	**0.638**
Registration	0.242	-0.101	**0.574**

The factor analysis of the patients with AD identified two factors in the MMSE-2 that explained approximately 45.3% of the total variance (Table 13). Factor 1 included five subtests (language, processing speed, drawing, attention and calculation, and registration) that explained 30.2% of the variance. We named this factor “tests for cognitive domains except for verbal memory”. Factor 2 included four subtests (orientation to time, recall, story memory, and orientation to place) that explained 15.1% of the variance. We named this factor “tests for episodic memory”.

**Table 13:** Factor analysis after Varimax rotation for the patients with Alzheimer’s disease.

Variables	Factors	
	1	2
Language	**0.749**	0.129
Processing speed	**0.718**	0.330
Drawing	**0.674**	-0.219
Attention and Calculation	**0.606**	0.305
Registration	**0.551**	-0.036
Orientation to time	0.233	**0.780**
Recall	-0.175	**0.612**
Story memory	0.020	**0.546**
Orientation to place	0.324	**0.477**

#### Concurrent validity

The concurrent validity of the MMSE-2 was examined through correlation with the values of the MMSE, the CDR, the CDR-SOB, the SVLT, the copy of RCFT, the SWF, the PWF, the Stroop Color-Word test, the K-BNT, and the digit span test (forward & backward). The results showed that the three versions of the MMSE-2 were significantly correlated with the cognitive function tests (Table 14). Particularly, the correlation coefficients were high between the MMSE-2:BV and the MMSE (r = 0.84, p<0.01), the MMSE-2:SV and the MMSE (r = 0.92, p<0.01), and the MMSE-2:EV and the MMSE (r = 0.83, p<0.01).

**Table 14:** Correlation between the MMSE-2 and cognitive measures.

	1	2	3	4	5	6	7	8	9	10	11	12	13	14	15
1. MMSE-2:BV	1														
2. MMSE-2:SV	0.9008*	1													
3. MMSE-2:EV	0.791*	0.875*	1												
4. MMSE	0.838*	0.920*	0.827*	1											
5. Digit-span- forward	0.345*	0.492*	0.529*	0.480*	1										
6. Digit-span- backward	0.37*	0.497*	0.561*	0.521*	0.488*	1									
7. SVLT-immediate recall	0.617*	0.655*	0.718*	0.642*	0.340*	0.410*	1								
8. SVLT-delayed recall	0.620*	0.598*	0.686*	0.586*	0.219*	0.362*	0.780*	1							
9. SVLT-recognition	0.645*	0.594*	0.618*	0.572*	0.276*	0.297*	0.652*	0.703*	1						
10. RCFT-copy	0.492*	0.607*	0.602*	0.602*	0.350*	0.438*	0.463*	0.398*	0.315*	1					
11. SWF-animal	0.496*	0.550*	0.600*	0.549*	0.347*	0.374*	0.602*	0.565*	0.470*	0.453*	1				
12. SWF-supermarket items	0.537*	0.545*	0.624*	0.535*	0.255*	0.373*	0.610*	0.584*	0.495*	0.391*	0.655*	1			
13. PWF	0.373*	0.486*	0.575*	0.500*	0.471*	0.493*	0.452*	0.383*	0.370*	0.459*	0.579*	0.512*	1		
14. Stroop Color-Word (word reading)	0.377*	0.471*	0.423*	0.487*	0.284*	0.272*	0.243*	0.216*	0.224*	0.433*	0.311*	0.286*	0.336*	1	
15. Stroop Color-Word (color naming)	0.562*	0.610*	0.675*	0.625*	0.357*	0.444*	0.638*	0.594*	0.491*	0.452*	0.581*	0.591*	0.493*	0.282*	1
16. K-BNT	0.543*	0.617*	0.666*	0.643*	0.424*	0.416*	0.580*	0.531*	0.553*	0.515*	0.567*	0.478*	0.453*	0.346*	0.519*

Abbreviations: MMSE-2, Mini-Mental State Examination; BV, Brief version; SV, Standard version; EV, Expanded version; MMSE, Korean version of Mini-Mental State Examination; SVLT, Seoul Verbal Learning Test; RCFT, Rey Complex Figure Test; SWF, Semantic Word Fluency; PWF, Phonemic Word Fluency; K-BNT, Korean version of Boston Naming Test.

Note.

*p<0.01.

#### Discriminant validity by CDR stage analysis

To examine the utility of the MMSE-2 to detect dementia severity, the participants were reclassified into five groups according to their CDR and CDR-SOB scores. Specifically, the healthy older adults were assigned a CDR score of 0 (CDR-SOB 0), the patients with MCI were assigned a CDR score of 0.5 (CDR-SOB 0.5–2.5), the patients with early stage of AD were assigned a CDR score of 0.5 (CDR-SOB 3.0–4.0), the patients with mild stage of AD were assigned a CDR score of 1 (CDR-SOB 4.5–9.0), and the patients with moderate stage of AD were assigned a CDR score of 2 (CDR-SOB 9.5–15.5). The average age, educational level, and gender of the participants are presented in Table 15. Although there was no significant difference in gender, **χ**^2^(1,414) = 3.03, *p* = 0.93, between the five groups, there were significant differences in age, F(4,409) = 14.703, *p*<0.001, and education, F(4,409) = 2.598, *p* = 0.036.

**Table 15:** Participants’ average age, education, and gender classified by CDR & CDR-SOB stage (M±SD).

	C(91)	MCI(226)	EAD(35)	MiAD(55)	MoAD(7)
Age	67.05±7.47	71.05±7.729	76.00±6.329	75.60±7.976	70.57±9.778
Education	10.98±5.207	11.45±4.976	9.97±5.025	9.164±5.186	11.57±5.653
Male/Female	29/62	90/135	14/21	20/35	2/5

Abbreviations: CDR, Clinical Dementia Rating; CDR-SOB, Clinical Dementia Rating-Sum of Boxes; M, Mean; SD, Standard deviation; C, Control; MCI, Mild Cognitive Impairment; EAD, Early stage of Alzheimer’s Disease; MiAD, Mild stage of Alzheimer’s Disease; MoAD, Moderate stage of Alzheimer’s Disease.

The scores of all three versions of the MMSE-2 for the participants in the five groups are presented in Table 16. An ANCOVA that controlled for age and education revealed significant differences between the five groups in all three versions of the MMSE-2. According to Tukey’s *post hoc* analyses, the five groups differed significantly with respect to the scores of the MMSE-2:BV and the MMSE-2:SV. However, on the MMSE-2:EV, the three groups (MCI, early stage of AD, and healthy older adults) differed significantly, but there was no significant difference between the patients with mild stage of AD and the patients with moderate stage of AD.

**Table 16:** The results of the three versions of the MMSE-2 in the five groups according to CDR & CDR-SOB (M±SD).

MMSE-2	C	MCI	EAD	MiAD	MoAD	F	*df*	*Post-hoc*
BV	14.43±1.33	13.37±1.58	10.71±1.86	8.13±2.28	6.00±1.53	161.69*	4, 407	C>MC>E>Mi>Mo
SV	27.26±2.66	25.71±2.35	22.03±2.85	18.35±3.50	14.43±3.60	151.66*	4, 407	C>MC>E>Mi>Mo
EV	49.84±9.59	43.48±7.81	33.14±5.78	27.71±6.45	19.29±5.68	105.77*	4, 407	C>MC>E>Mi = Mo

Abbreviations: M, Mean; SD, Standard deviation; C, Control; MCI, Mild Cognitive Impairment; EAD, Early stage of Alzheimer’s Disease; MiAD, Mild stage of Alzheimer’s Disease; MoAD, Moderate stage of Alzheimer’s Disease.

Note.

*p<0.01.

### Diagnostic utility

To measure the diagnostic utility of the three versions of the MMSE-2, the ROC curve analysis and the area under the curve (AUC) was calculated. The results of each version of the MMSE-2 were as follows.

#### MMSE-2:BV

**Fig 1:**
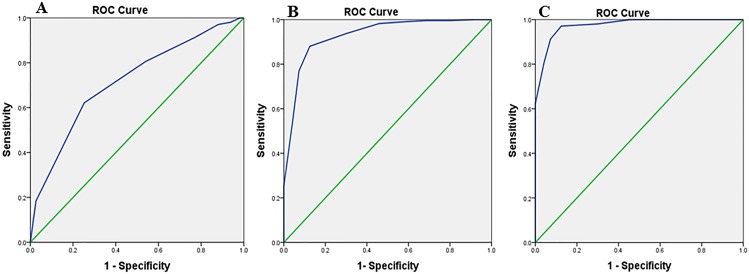
MMSE-2:BV. **Receiver Operator Characteristic (ROC) curve analysis of the MMSE-2: Brief version in the three groups.** (A) Normal vs. MCI, Area Under the Curve (AUC) = 0.71. (B) MCI vs. AD, Area Under the Curve (AUC) = 0.93. (C) Normal vs. AD, Area Under the Curve (AUC) = 0.97.

First, for discriminating the healthy older adults from the patients with MCI, the AUC of the MMSE-2:BV was 0.71 (95% confidence interval, CI, 0.64–0.77, *p*<0.001). The sensitivity of the MMSE-2:BV was 60% and the specificity was 75% when using a cut-off score of ≤ 14 of 16 to predict MCI. Second, for discriminating the patients with MCI from the patients with AD, the AUC of the MMSE-2:BV was 0.93 (95% CI, 0.90–0.96, *p*<0.001). The sensitivity of the MMSE-2:BV was 88% and the specificity was 87% when using a cut-off score of ≤ 10 of 16 to predict AD. Finally, for discriminating the healthy older adults from the patients with AD, the AUC of the MMSE-2:BV was 0.97 (95% CI, 0.96–0.99, *p*<0.001). The sensitivity of the MMSE-2:BV was 98% and the specificity was 70% when using a cut-off score of ≤ 10 of 16 to predict AD (Fig 1).

#### MMSE-2:SV

**Fig 2:**
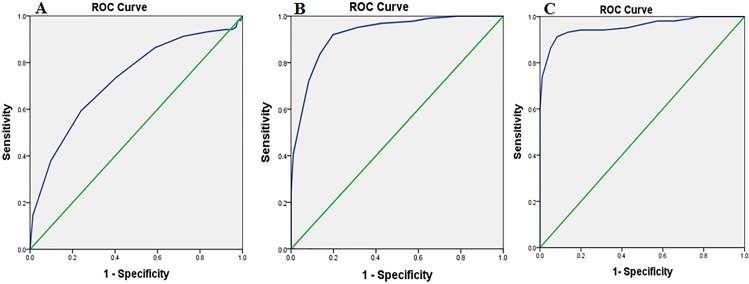
MMSE-2:SV. **Receiver Operator Characteristic (ROC) curve analysis of the MMSE-2: Standard version in the three groups.** (A) Normal vs. MCI, Area Under the Curve (AUC) = 0.72. (B) MCI vs. AD, Area Under the Curve (AUC) = 0.93. (C) Normal vs. AD, Area Under the Curve (AUC) = 0.95.

First, for discriminating the healthy older adults from the patients with MCI, the AUC of the MMSE-2: SV was 0.72 (95% CI, 0.66–0.79, *p*<0.001). The sensitivity of the MMSE-2:SV was 74% and the specificity was 59% when using a cut-off score of ≤ 26 of 30 to predict MCI. Second, for discriminating the patients with MCI from the patients with AD, the AUC of the MMSE-2:SV was 0.93 (95% CI, 0.89–0.96, *p*<0.001). The sensitivity of the MMSE-2:SV was 84% and the specificity was 87% when using a cut-off score of ≤23 of 30 to predict AD. Finally, for discriminating the healthy older adults from the patients with AD, the AUC of the MMSE-2:SV was 0.95 (95% CI, 0.92–0.98, *p*<0.001). The sensitivity of the MMSE-2:SV was 92% and the specificity was 87% when using a cut-off score of ≤ 23 of 30 to predict AD (Fig 2).

#### MMSE-2:EV

**Fig 3:**
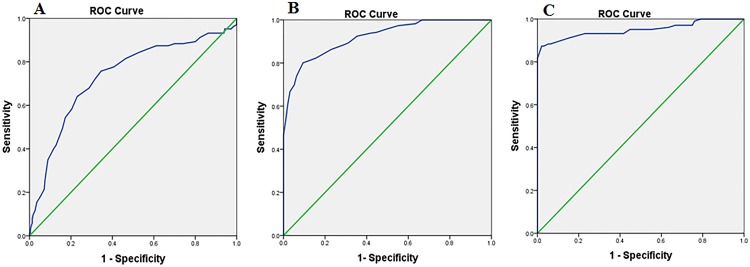
MMSE-2:EV. **Receiver Operator Characteristic (ROC) curve analysis of the MMSE-2: Expanded version in the three groups.** (A) Normal vs. MCI, Area Under the Curve (AUC) = 0.73. (B) MCI vs. AD, Area Under the Curve (AUC) = 0.92. (C) Normal vs. AD, Area Under the Curve (AUC) = 0.94.

First, for discriminating the healthy older adults from the patients with MCI, the AUC of the MMSE-2:EV was 0.73 (95% CI, 0.66–0.80, *p*<0.001). The sensitivity of the MMSE-2:EV was 71% and the specificity was 69% when using a cut-off score of ≤ 46 of 90 to predict MCI. Second, for discriminating the patients with MCI from the patients with AD, the AUC of the MMSE-2:EV was 0.92 (95% CI, 0.89–0.95, *p*<0.001). The sensitivity of the MMSE-2:EV was 82% and the specificity was 85% when using a cut-off score of ≤ 36 of 90 to predict AD. Finally, for discriminating the healthy older adults from the patients with AD, the AUC of the MMSE-2:EV was 0.94 (95% CI, 0.91–0.98, *p*<0.001). The sensitivity of the MMSE-2:EV was 92% and the specificity was 71% when using a cut-off score of ≤ 34 of 90 to predict AD (Fig 3).

## Discussion

This study verified the newly developed MMSE-2 as a reliable and valid cognitive screening measure for MCI and AD in a Korean population. The results demonstrated several key points.

First, the results of the MMSE-2 and other neuropsychological assessments that measure attention, verbal memory, visuospatial function, language function, and frontal/executive function significantly differed between the three groups (healthy older adults, MCI, AD).

Second, the MMSE-2 was shown to have good internal consistency, high test-retest reliability, and high inter-rater reliability.

Third, to demonstrate the construct validity of the MMSE-2, a factor analysis was performed on each of the three groups. For the patients with MCI, the MMSE-2 was divided into three factors. The first factor included the tests related to working memory and frontal lobe functioning, such as attention and calculation, drawing, and psychomotor speed task. The drawing test was correlated with temporal elements of working memory, and it had indirect effects on attention and calculation [26]. The second factor included the tests related to verbal memory, such as recall and story memory tests. The third factor included the tests related to orientation and immediate recall, such as orientation to place, orientation to time, and registration tests. Tests of working memory and verbal memory were sensitive for detecting early decline in cognitive function [27–29]. Moreover, working memory was related to a decline in episodic memory, and tests of verbal memory measured episodic memory. Thus, the tests related to episodic memory may be sensitive in assessing cognitive functioning of the patients with MCI.

For the patients with AD, the MMSE-2 was divided into two factors. The first factor included language, processing speed, drawing, attention and calculation, and registration, which can measure overall cognitive functioning other than memory. The second factor included the tests related to episodic memory, such as orientation to time, recall, story memory, and orientation to place.

We compared the factor analysis of the patients with AD and that of the patients with MCI. For the patients with MCI, the tests of orientation to time and orientation to place were not sensitive to changes in cognitive functions, but for the patients with AD, tests of recall and story memory and tests of orientation to time and orientation to place were sensitive to changes in cognitive functions. Therefore, as mild cognitive impairment progresses to AD, tests related to episodic memory seems to become sensitive to changes in cognitive function.

In the healthy older control group, the MMSE-2 was divided into two factors: tests that are sensitive to decline in cognitive functions, such as recall, orientation to place, story memory, processing speed, attention and calculation, and orientation to time orientation; and tests that are not sensitive to decline in cognitive functions, such as registration, language and drawing. Therefore, this demonstrated that the factor analyses differed between the groups based on the degree of cognitive impairment and confirmed that all MMSE-2 items were clearly divided between the groups.

The present study also showed that the MMSE-2 was highly correlated with various neuropsychological assessments with verified validity. Particularly, the MMSE-2 had a very high correlation with the MMSE, and it also demonstrated a high correlation with verbal memory frontal lobe function tests. Even though there is executive function test in the MMSE such as attention and calculation, the MMSE is insensitive to impairments in executive functioning, abstract reasoning, and visual perception/concentration [10]. However, the MMSE-2, as Folstein et al. [12] suggested, has shown its ability to measure executive function in more detail, and thus, it can measure a greater variety of cognitive functions than the MMSE.

Fourth, the scores of the MMSE-2 could also discriminate between each of the CDR and CDR-SOB stages. Thus, the scores of the MMSE-2 declined significantly as CDR and CDR-SOB scores increased, which confirms that the MMSE-2 is able to discriminate between the stages of CDR and CDR-SOB. This showed that the MMSE-2 is a useful instrument as a screening measure for detecting the progress of cognitive impairment. However, with the MMSE-2:EV, there was no significant difference between the patients with mild stage of AD and the patients with moderate stage of AD. One of many possible reasons for this finding is that the difficulty levels of story memory and processing speed tests might seem too challenging for the patients beyond mild stage of AD, and so a floor effect is highly probable. Thus, the MMSE-2:BV and MMSE-2:SV can be more effective than the MMSE-2:EV in assessing cognitive functions of the patients with mild stage of AD and the patients with moderate stage of AD.

Finally, the sensitivity and specificity of the three versions of the MMSE-2 in discriminating between the healthy older adults and the patients with MCI were tested: for the MMSE-2:BV, the sensitivity was 60% and the specificity was 75% at the cut-off score of 14/15; for the MMSE-2:SV, the sensitivity was 74% and the specificity was 59% at the cut-off score of 26/27; and for the MMSE-2:EV, the sensitivity was 71% and the specificity was 69% at the cut-off score of 46/47. All three versions of the MMSE-2 could similarly discriminate between the two groups.

Moreover, the sensitivity and specificity of the three versions of the MMSE-2 in discriminating between the patients with MCI and the patients with AD were tested: for the MMSE-2:BV, the sensitivity was 88% and the specificity was 87% at the cut-off score of 11/12; for the MMSE-2:SV, the sensitivity was 84%, and the specificity was 87% at the cut-off score of 23/24; and for the MMSE-2:EV, the sensitivity was 82% and the specificity was 85% at the cut-off score of 36/37. All three versions of the MMSE-2 could similarly discriminate between the two groups.

The sensitivity and specificity of three versions of the MMSE-2 in discriminating between the healthy older adults and the patients with AD were tested: for the MMSE-2:BV, the sensitivity was 98% and the specificity was 70% at the cut-off score of 10/11; for the MMSE-2:SV, the sensitivity was 93%, and the specificity was 80% at the cut-off score of 22/23; and for the MMSE-2:EV, the sensitivity was 92% and the specificity was 71% at the cut-off score of 34/35. All three versions of the MMSE-2 could similarly discriminate between the two groups.

Overall, the MMSE-2 is useful for discriminating between the patients with MCI and the patients with AD and between healthy older adults and the patients with AD, but its ability to discriminate between the healthy older adults and the patients with MCI is less than satisfactory. Nevertheless, the MMSE-2 is slightly more sensitive in this area than the MMSE, which has sensitivity of 82.7% at the cut-off score of 23/24 [9].

In summary, according to these results, as Folstein et al. [12] suggested, the MMSE-2 can be used as a valid and reliable screening measure for assessing cognitive impairment in clinical settings in a Korean population, but its ability to distinguish the patients with MCI from healthy older adults may not be as highly sensitive as expected.
